# Supplementary material for: Viscosity-Sensitive Solvatochromic Fluorescent Probes for Lipid Droplets Staining
Source: Biosensors (Basel). 2022 Oct 9;12(10):851. doi: 10.3390/bios12100851 (PMC9599285; doi:10.3390/bios12100851)
Supplement: Supplementary file 1 [file biosensors-12-00851-s001.zip › biosensors-1936421-supplementary.pdf]

Supporting Information

1. Materials and Instruments

NMR spectra were examined from AVANCE II 400 MHz Digital NMR Spectrometer with TMS as an internal standard; electronic absorption spectra were recorded on a LabTech UV Power spectrometer; fluorescence spectra were obtained with a HITACHI F4600 fluorescence spectrophotometer; the fluorescent images of the cells were obtained with Leica SP8 inverted fluorescence confocal microscope. Analysis was exhibited on silica gel plates and column chromatography was carried out over silica gel (mesh 200-300). Both TLC and silica gel were purchased from the Qingdao Ocean Chemicals.

2. General Information for Spectroscopic Studies

A certain amount of probe **Couoxo-LD** was dissolved in dimethyl sulfoxide (DMSO) to obtain the stock solution (1 mg/mL). The 10 μg/mL probe was used in all spectroscopic experiments and prepared by adding 20 μL of the probe stock solution to the cuvette after dilution to 2 mL with different solvents. Solutions of various interfering substances (10 μM) were prepared in ultrapure water. The PBS buffer solutions with different pH from 1-14 were measured and prepared with a pH meter. The excitation wavelength was 510 nm; Vol. 600 V; both of the excitation and emission slit widths are 5 nm.

3. Cell Culture and Cytotoxicity Assays

Hela cells were provided by Jiangsu Kaiji Biotechnology Co., Ltd. The living HeLa cells were cultured in the Dulbecco's modified Eagle's medium (DMEM) and supplemented with fetal bovine serum (10% FBS) under atmosphere containing 5% CO_2 and 95% air at 37 °C.

4. Imaging of HeLa cells And Co-Localization Experiments

The concentration of the probe in the cell imaging experiments was 10 μg/mL, and the cells were incubated at a temperature of 37°C with 5% CO_2 for 30 min. To remove the residual probe, the cells were rinsed three times with PBS buffer solution before imaging. Starvation cell group: obtained by feeding with fetal bovine serum (10%, FBS) only without any added high sugar for 5h under the same culture conditions. Dynamic lipid droplet imaging: starved cultured cell groups were first co-incubated with probe **Couoxo-LD** (10 μg/mL) for 30 min and then scanned for imaging at different time ranges after adding oleic acid (10 μM) stimulation. Finally, the cells were imaged with a Leica SP8 inverted fluorescence confocal microscope with excitation wavelength of 405 nm and emission wavelengths of 480-510 nm (green channel) and 600-650 nm (red channel).

5. Imaging of the Probe Couoxo-LD in Living Zebrafish

Live zebrafish or embryos were incubated with 10 μM **Couoxo-LD** probe in water for 1 h, then transferred to another imaging plate containing trace water, and placed under confocal microscope for imaging with excitation wavelength of 405 nm.

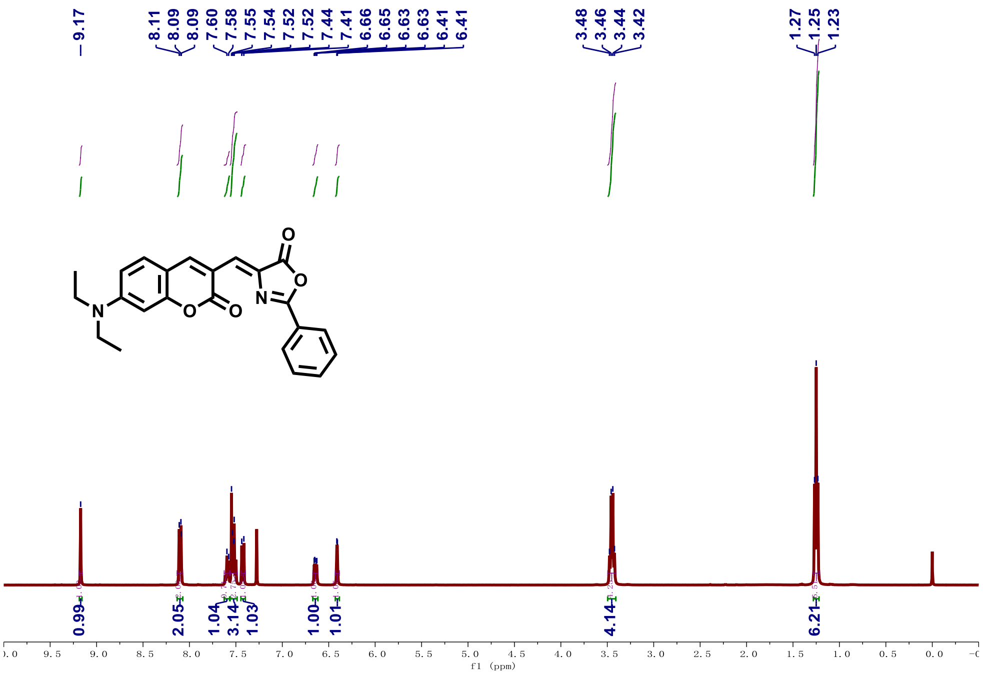

Figure S1. ^{1}H NMR ($CDCl_3$) spectrum of **Couoxo-LD**.

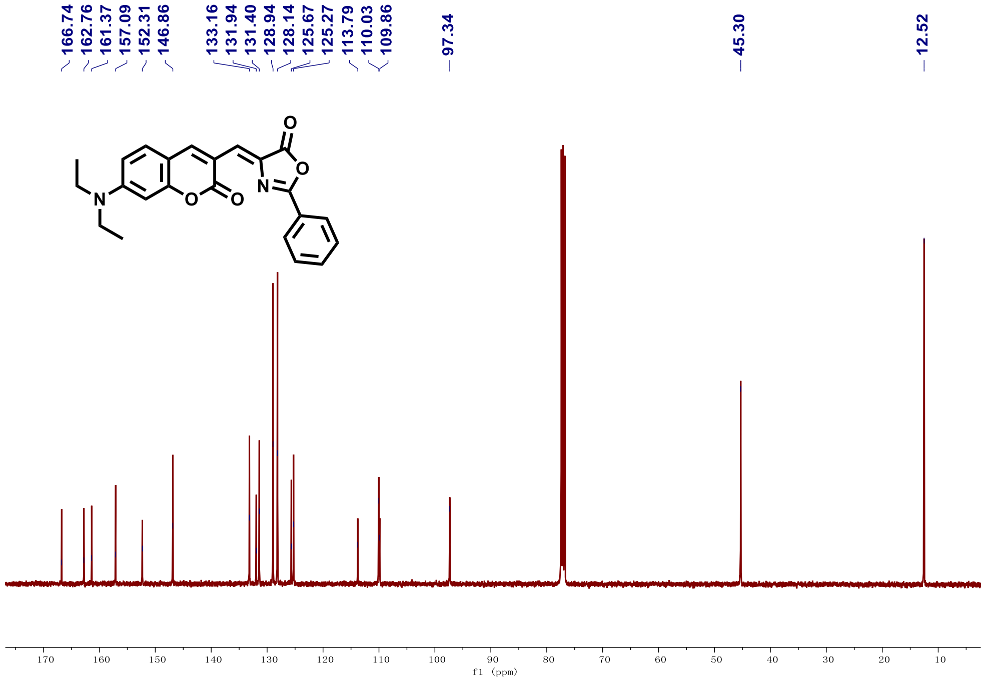

Figure S2. ^{13}C NMR ($CDCl_3$) spectrum of **Couoxo-LD**.

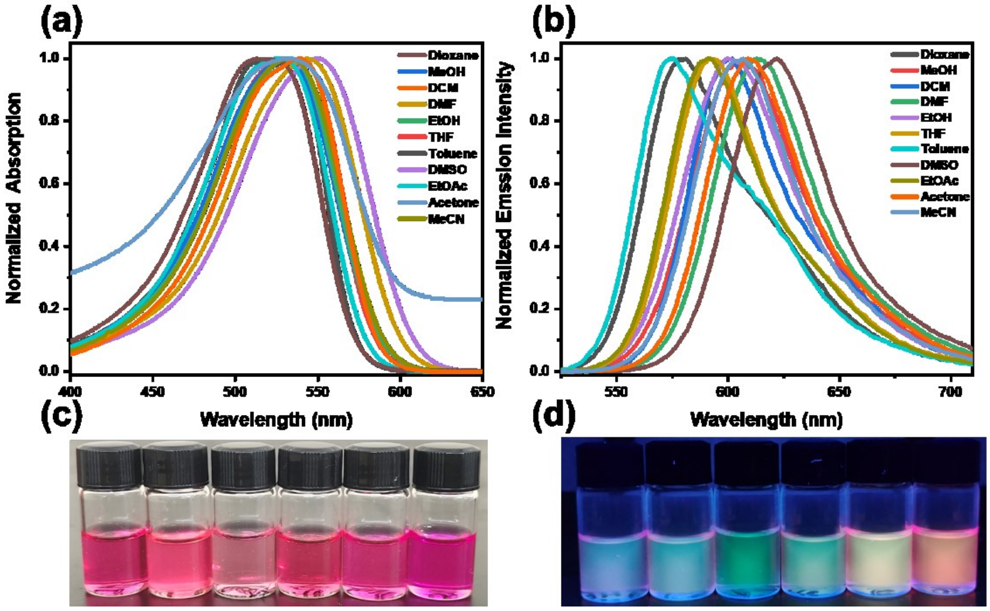

Figure S3. Normalized (a) absorption and (b) fluorescence spectra of **Couxox-LD** (10 μM, λ_{ex} = 510 nm) in different solvents. (c) and (d) are photographs of **Couoxo-LD** (10 μM) in different solvents under natural light and 365 nm handheld UV lamp irradiation, respectively.

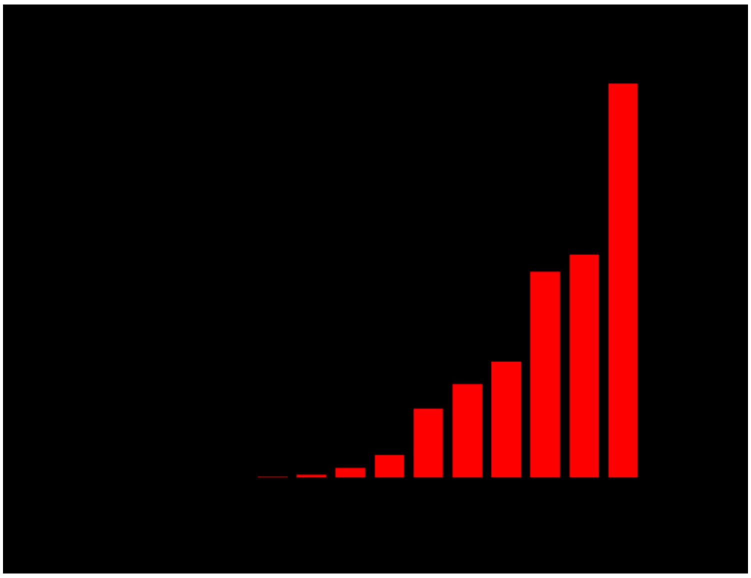

Figure S4. Maximum emission wavelength of **Couoxo-LD** (10 μM, λ_{ex} = 510 nm) versus liquid viscosity (PBS buffers–glycerol) system.

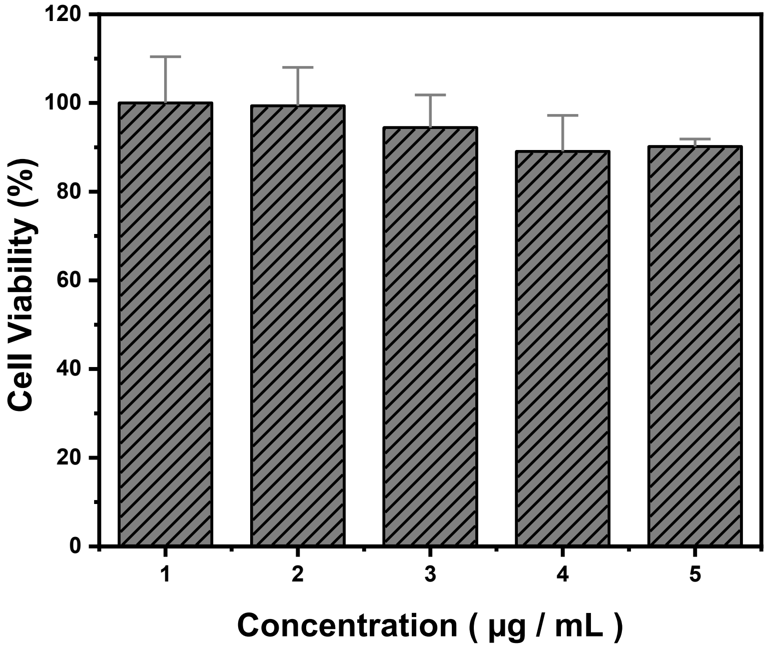

Figure S5. Cytotoxicity assays of probe **Couoxo-LD** at different concentrations (0 μM; 2 μM; 5 μM; 10 μM; 20 μM; 30 μM) for HeLa cells.

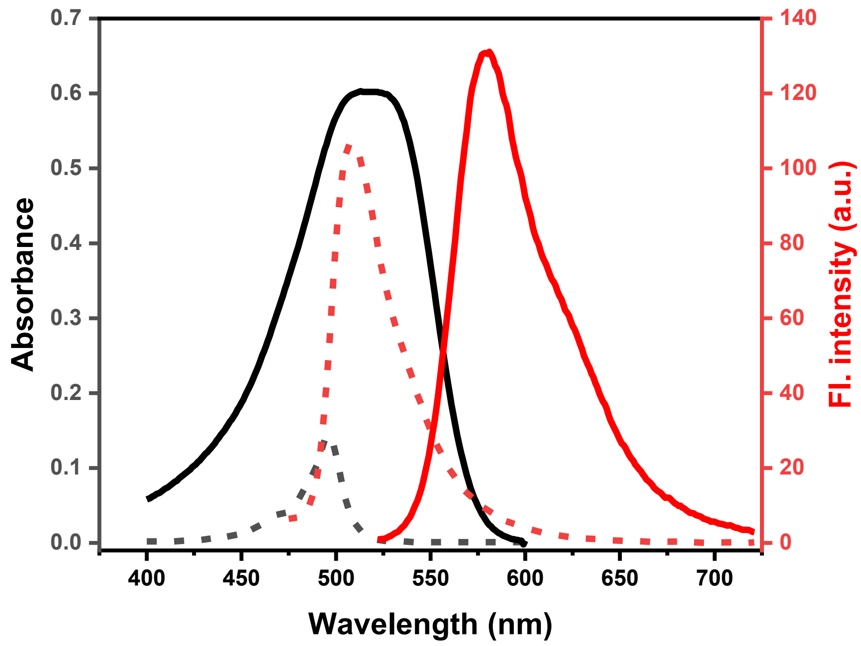

Figure S6. Absorption and emission spectra of **Couoxo-LD** (solid line) and BODIPY (dashed line) in dioxane.

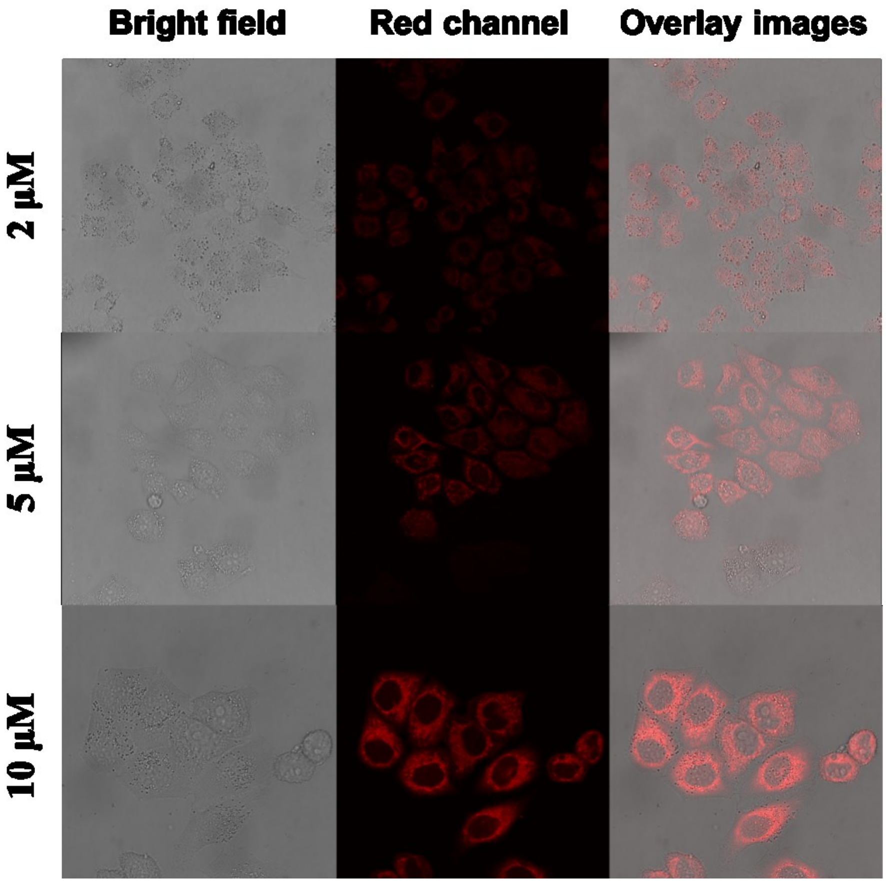

Figure S7. Fluorescence images of live HeLa cells incubated with different concentrations of **Couoxo-LD** at 37 ˚C for 30 minutes.

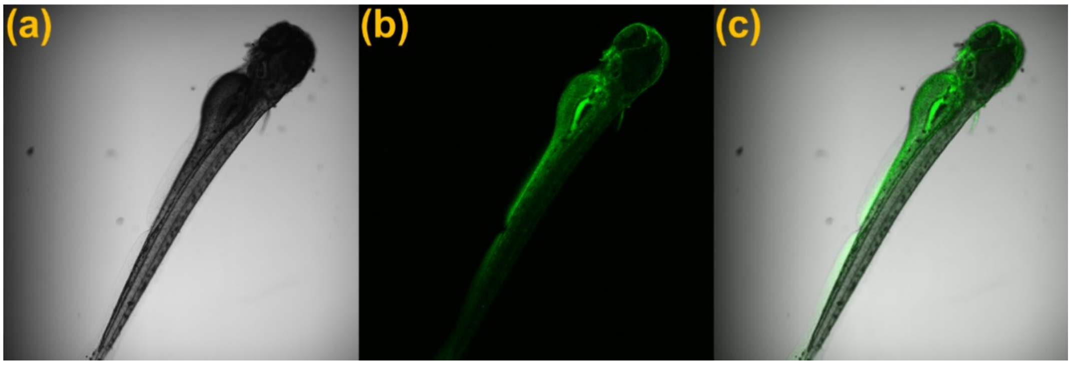

Figure S8. Fluorescence images of live zebrafish treated with BODIPY (5 μM). (a) Bright-field view; (b) green channel, λ_{ex} = 405 nm, λ_{em} = 480–510 nm; (c) merged images. Scale bar. 500 μm.
